# A Comparative Analysis of *CD32A* and *CD16A* Polymorphisms in Relation to Autoimmune Responses in Pemphigus Diseases and Subepithelial Autoimmune Blistering Disorders

**DOI:** 10.3390/genes11040371

**Published:** 2020-03-30

**Authors:** Justyna Gornowicz-Porowska, Michał J. Kowalczyk, Agnieszka Seraszek-Jaros, Monika Bowszyc-Dmochowska, Elżbieta Kaczmarek, Ryszard Żaba, Marian Dmochowski

**Affiliations:** 1 Department of Medicinal and Cosmetic Natural Products, Poznan University of Medical Sciences, Mazowiecka 33 Street, 60-623 Poznań, Poland; 2 Autoimmune Blistering Dermatoses Section, Department of Dermatology, Poznan University of Medical Sciences, Przybyszewskiego 49 Street, 60-355 Poznań, Poland; mkdmoch@wp.pl; 3 Department of Dermatology and Venereology, Poznan University of Medical Sciences, Przybyszewskiego 49 Street, 60-355 Poznań, Poland; ryszardzaba@gmail.com; 4 Department of Bioinformatics and Computational Biology, Poznan University of Medical Sciences, Rokietnicka 4 Street, 60-806 Poznań, Poland; agnetpa@gmail.com (A.S.-J.); elka@ump.edu.pl (E.K.); 5 Cutaneous Histopathology and Immunopathology Section, Department of Dermatology, Poznan University of Medical Sciences, Przybyszewskiego 49 Steet, 60-355 Poznań, Poland; m.bowdmo@wp.pl

**Keywords:** pemphigus, bullous pemphigoid, Fc receptors, autoantibodies, polymorphism

## Abstract

Autoimmune blistering dermatoses (ABDs) are characterized by autoantibodies to keratinocyte surface antigens and molecules within the dermal–epidermal junction causing disruption of skin integrity. The affinity of Fc receptors (FcRs) causing an autoimmune response in ABDs may vary based on single-nucleotide polymorphisms (SNPs) in FcRs determining the course of disease. This study aimed to explore the effects of *CD16A* and *CD32A* SNPs on the autoimmune response in several ABDs. In total, 61 ABDs patients were investigated. ELISA tests, direct immunofluorescence (DIF), TaqMan SNP Genotyping Assays, and statistical analyses were performed. The CA genotype (composed of allele C and A) of rs396991 in *CD16A* had a higher affinity for tissue-bound IgG1 in pemphigus and for C3 in subepithelial ABDs, showing statistical significance. The greatest relative risk (odds ratio) was reported for AA (rs396991 of *CD16A*) and CC (rs1801274 of *CD32A*) homozygotes. There were no statistically significant differences between certain genotypes and specific circulating autoantibodies (anti-DSG1, anti-DSG3 IgG in pemphigus; anti-BP180, anti-BP230 IgG) in subepithelial ABDs. Our findings indicated that rs396991 in *CD16A* may be of greater importance in ABDs development. Moreover, FcR polymorphisms appeared to have a greater impact on tissue-bound antibodies detected using DIF than circulating serum antibodies in ABDs.

## 1. Introduction

Pemphigus and subepithelial autoimmune blistering disorders (SABDs), including bullous pemphigoid (BP), represent a rare heterogeneous group of autoimmune blistering dermatoses (ABDs) [1,2]. Histopathological findings show intraepidermal clefts in pemphigus and subepithelial separation in SABDs. They are clinically characterized by the presence of cutaneous and/or mucosal blisters and their evolutionary lesions. Pemphigus and BP are identified by both circulating and tissue-bound autoantibodies against desmosomal cadherins (mainly desmoglein 1 and 3 (DSG1/3) in pemphigus) and dermal–epidermal junction components (mainly hemidesmosomal proteins, e.g., BP180 and BP230 in BP) [1,2,3]. Autoantibodies detected in pemphigus and BP usually belong to the IgG4 subclass of IgG, reflecting the active stage of disease [4] through the Th2 immune response mechanism [5]. Precise molecular mechanisms leading to blister development in ABDs remain undiscovered. ABDs are associated with the destruction of numerous components of the dermal–epidermal junction and/or desmosomal components [2]. Thus, proteolytic enzymes (e.g., neutrophil elastase) are involved in a multitude of physiological reactions and may impact dermal–epidermal and desmosomal integrity. On the other side, Fc receptors (FcRs) are capable of modulating the immune response by playing an important role in the etiopathogenesis of autoimmune diseases [6]. Circulating neutrophils from patients with ABDs were proven to stimulate inflammatory cells (priming), a phenomenon resulting from enhanced function of FcRs causing an increased ability to bind antibodies, presumably resulting in tissue damage.

Human FcRs are probably associated with the development or progression of various autoimmune diseases (e.g., rheumatoid arthritis, systemic lupus erythematosus) [7,8,9,10,11]. Experimental models of ABDs showed FcR involvement, and it is currently suspected that these receptors influence their pathogenesis [12]. FcRs play key functions in the specific regulation (both up and downregulation) of immune responses that could result in skin tissue damage [6,7].

There is strong evidence indicating special engagement of IgG-binding FcRs (FcγRs) in the pemphigus group of diseases and bullous pemphigoid (BP) [9,12]. FcγRs include FcγRI (CD64), FcγRII (CD32) and FcγRIII (CD16). Moreover, CD32 exists in two major isoforms, A and B, with activating and inhibitory functions, respectively [13].

It was postulated that FcR polymorphisms affect the affinity of immunoglobulin molecules that determine the susceptibility and clinical phenotype of autoimmune and inflammatory disorders [14,15,16,17], such as pemphigus and/or SABDs [18,19,20]. However, to the best of our knowledge, little molecular data exists regarding gene mutations associated with the role of FcRs in both pemphigus and SABDs. As far as the functional relevance and disease associations of single-nucleotide polymorphisms (SNPs) are concerned, rs1801274 is associated with higher affinity for human IgG and may be related to Kawasaki disease, childhood idiopathic thrombocytopenic purpura and systemic lupus erythematosus (SLE), and rs396991 is associated with higher affinity for human IgG and may be related to susceptibility to ulcerative colitis, SLE, rheumatoid arthritis and idiopathic thrombocytopenic purpura [21,22].

Hence, we attempted to identify genetic contributions of FcγRs to both prognosis and susceptibility to pemphigus and SABDs. Based on our previous findings [9] on the protein expression level of FcRs in ABDs, we selected the most promising candidates to examine gene polymorphisms. Haplotypes of *CD32A* and *CD16A/B* polymorphic variants were postulated to influence IgG-mediated responses in neutrophils [23]. rs1801274 and rs396911 polymorphisms influence the affinity of different IgG subclasses [19]. A nonsynonymous polymorphism (519G > A, rs1801274) in exon 4 encoding the membrane proximal Ig-like domain of *CD32A* leads to an arginine (R) to histidine (H) change at position 131 and modifies receptor affinity for ligands. The alternation in ligand affinity is associated with functional relevance in determining cellular interactions with IgG antibodies, including the clearance of IgG2 immune complexes [6]. In *CD16A*, a point substitution of T to G at nucleotide 559 (rs396991) alters the phenylalanine (F) at amino acid position 158 to valine (V). The FcγRIIIa-158V exhibits higher affinity for IgG1 and IgG3 relative to the 158F (176F) allele [6].

The aim of this study was to identify and evaluate the possible association between the expression of certain SNPs of *CD16A* and *CD32A* genes alongside systemic and local immune responses in ABDs, with a focus on rs396991 of the *CD16A* gene and rs1801274 of the *CD32A* gene in pemphigus and SABDs.

## 2. Materials and Methods

This study was approved by a local bioethical committee (Poznan University of Medical Sciences, 953/14, Poland, 2014). In total, 61 patients with ABDs were examined before treatment initiation, including 47 SABD patients (28 females, 19 males, mean age 77.5; 39 BP, 4 mucous membrane pemphigoid (MMP), 2 epidermolysis bullosa acquisita (EBA) and 2 Brunsting–Perry-type pemphigoid) and 14 pemphigus patients (9 females, 5 males, mean age 63.5; 6 pemphigus vulgaris (PV) and 8 pemphigus foliaceus (PF)). Patients with SABDs, sharing the subepithelial localization of tissue separation as a key pathological feature, and patients with pemphigus diseases, sharing the intraepithelial localization of tissue separation as a key pathological feature, served as the controls for each other.

The detailed characteristics of the examined groups are presented in Table 1.

### 2.1. Sample Collection and Tissue Specimens

The examined material consisted of perilesional skin tissues, sera and ethylenediaminetetraacetic acid (EDTA)-aspired whole blood. Patients were diagnosed and treated at the Department of Dermatology, Poznan University of Medical Sciences in Poland. The inclusion criteria involved (i) the clinical picture, (ii) a positive direct immunofluorescence (DIF) test of perilesional skin for ABDs and (iii) histological features of ABDs. When needed, some cases were corroborated with a multi-analyte ELISA (DSG1, DSG3, BP180, BP230, envoplakin, type VII collagen) and an indirect immunofluorescence mosaic for IgG, IgG1 and IgG4 antibodies to three laminin-332 epitopes.

**Figure 1:**
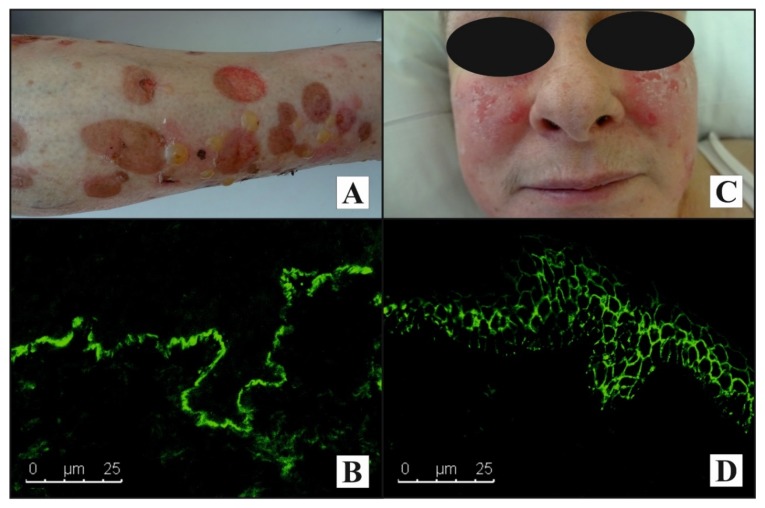
A representative elderly female with bullous pemphigoid homozygous for allele A in rs396991 and heterozygous TC in rs1801274: (**A**) Numerous blisters and their evolutionary lesions on the shin; (**B**) linear IgG4 deposits along the dermal–epidermal junction detected by direct immunofluorescence visualized with laser-scanning confocal microscopy. A mono-analyte ELISA test revealed elevated levels of anti-BP180 IgG (200 RU/mL, cut-off 20 RU/mL). A representative middle-aged female with pemphigus foliaceus homozygous for allele A in rs396991 and homozygous for allele T in rs1801274: (**C**) Widespread erythematous plaques and erosions with superficial scaling on the cheeks; (**D**) intercellular IgG4 deposits within epidermis detected by direct immunofluorescence visualized with laser-scanning confocal microscopy (original objective magnification ×40). A mono-analyte ELISA test revealed elevated levels of anti-DSG1 IgG (200 RU/mL, cut-off 20 RU/mL).

Representative patients with pemphigus and BP are presented in Figure 1.

Skin tissues were frozen and subjected to 4 μm sectioning, followed by mounting on poly-L-lysine coated glass slides.

Whole blood used in the serological and genetic tests was taken at the time of hospital admission/ambulatory care.

### 2.2. Direct Immunofluorescence and Microscopic Examination

DIF of perilesional skin was performed in all cases to detect IgA, IgG, IgG1, IgG4 and C3 deposits. The tissue sections were incubated in a humid chamber for 30 min at room temperature (RT) with commercially available fluorescein isothiocyanate (FITC)-conjugated anti-human IgA, IgM, IgG and C3 rabbit polyclonal antibodies (Dako, Denmark), and the FITC-conjugated anti-human IgG subclasses IgG1 and IgG4 murine monoclonal antibodies (Sigma, USA). The antibodies were used at a working dilution of 1:100 in phosphate buffer saline (PBS). The samples were then washed in PBS (pH 7.2) at room temperature for 15 min with gentle agitation. The slides were then coverslipped and examined. Skin samples were examined by up to three independent observers using up to three methods, including blue light-emitting diode technology-operated microscopy (EuroStar III Plus microscope, Euroimmun, Germany), short arc mercury lamp-operated microscopy (BX40, Olympus, Japan) and laser-scanning confocal microscopy (the ZEISS LSM510 system with Axiovert 200M laser-scanning confocal microscope, Carl Zeiss Jena GmbH, Germany) [24].

The intensities of the deposits on the slides were reported according to the arbitrarily assigned semiquantitative four-point scale (from “−“ to “+++”) at identical objective magnifications (×20 and ×40).

### 2.3. Immunoenzymatic Assay

Specific circulating serum IgG autoantibodies were detected with commercially available ELISAs. The results of quantitative measurements with ELISA kits utilizing recombinant specific antigens, i.e., DSG1, DSG3, BP180 and BP230 (Euroimmun, Germany), were used for statistical analyses. The manufacturer’s cut-off for determining the positive level of antibodies measured was 20 RU/mL for all tests (detection range 0–200 RU/mL). All measurements were made with the use of Asys Expert 96 ELISA plate reader equipped with Microwin 2000 software by a single experienced operator following the manufacturer’s instructions.

### 2.4. DNA Isolation and SNP Analysis/Genotyping

Genomic DNA was isolated from 200 μL of whole blood with the use of NucleoSpin Blood column-based kit according to the manufacturer’s instructions (cat. 740951, Macherey-Nagel, Germany). The concentration of the eluted gDNA was measured with the use of the NanoDrop system. Samples were then diluted to 20 ng/μL with 5 mM Tris/HCl.

Genotyping of rs1801274 and rs396991 was performed with the use of the TaqMan SNP Genotyping Assay (cat. 4351379, Applied Biosystems) and Fast Probe qPCR Master Mix 2× (cat. E0422, EURx, Poland). Real-time PCR reactions were performed using 20 μL of sample, consisting of 10 μL 2× master mix, 8.5 μL water, 0.5 μL 40× probe assay and 1.0 μL gDNA. The amplification was performed with the use of the LightCycler 2.0 capillary system (Roche Diagnostics, Germany). The PCR program was as follows: 10 min at 95 °C at least 40× (15 s at 92 °C, ramp rate (RR) 5 °C/s; 60 s at 60 °C, RR 5 °C/s, signal acquisition at 530 nm and 560 nm channels), with a final extension of 10 min at 60 °C. All reaction batches were cross-calibrated.

### 2.5. Statistical Analysis

Qualitative data were presented as numbers and percentages. χ^2^ and Fisher’s exact tests were used to compare groups. Quantitative data were presented by the mean, standard deviation (SD) and range. All quantitative results were first verified for normality (Shapiro–Wilk test). For comparison between two groups, a nonparametric Mann–Whitney U-test was used. Odds ratios (ORs) and 95% confidence intervals (CIs) were calculated. Deviations from Hardy–Weinberg equilibrium expectations were determined using the χ^2^ test. A *p* < 0.05 was arbitrarily considered statistically significant. Statistical analysis was performed using STATISTICA, version 13 (StatSoft, Inc, Tulsa, OK, USA).

## 3. Results

### 3.1. CD16A and CD32A Polymorphisms in Pemphigus and SABDs

The distribution of various alleles and genotypes of rs396991 of *CD16A* and rs1801274 of *CD32A* in the examined population of SABDs and pemphigus is shown in Table 2. The greatest relative risk (odds ratios) were reported for AA (rs396991 of *CD16A*) and CC (rs1801274 of *CD32A*) homozygotes (OR 1.62 and OR 2.29, respectively), which was statistically insignificant.

There was no statistically significant deviation from the Hardy–Weinberg equilibrium.

### 3.2. rs396991 of *CD16A* and rs1801274 of CD32A in Relation to Tissue-Bound Antibodies

Only two relations between rs396991 of *CD16A* (CA in SABDs and AA in pemphigus) and tissue-bound antibodies (IgG1, C3) with statistical significance were found.

There was a statistically significant association between the rs396991 CA genotype in *CD16A* and C3 immunoreactants detected by DIF in SABDs (*p* = 0.0078). Additionally, there was a statistically significant association between the rs396991 AA genotype in *CD16A* and IgG1 immunoreactants detected by DIF in pemphigus (*p* = 0.0121). Thus, levels of deposited C3 in SABDs and IgG1 in pemphigus seemed to differ based on the examined polymorphisms.

The comparison of tissue-bound antibodies (direct immunofluorescence results) in relation to specific genotypes of the examined polymorphisms in SABDs and pemphigus are presented in Table 3 and Table 4, respectively.

### 3.3. rs396991 of CD16A and rs1801274 of CD32A in Relation to Circulating Antibodies against DSG1/3, BP180 and BP230

No statistically significant differences between certain genotypes and specific autoantibodies detected in the serum were identified in either pemphigus or SABDs (*p* > 0.05).

The comparison of circulating anti-BP180 IgG and anti-BP230 IgG levels in relation to specific alleles of the examined polymorphisms in the SABDs population are presented in Table 5.

The comparison of circulating anti-DSG1 IgG and anti-DSG1 IgG levels in relation to specific alleles of examined polymorphisms (rs396991 and rs1801274) in the pemphigus population are presented in Table 6.

## 4. Discussion

In this work we examined if certain polymorphisms of two FcR genes, *CD16A* and *CD32A*, underlie the attachment of specific autoantibody types and abnormal blistering skin phenotypes in pemphigus and SABDs [19,20]. It was postulated that these polymorphisms affect receptor signaling and may be associated with ABDs susceptibility. Recent investigative efforts included the identification of mutations in genes encoding immunological molecules and their possible associations with pathological autoimmune response in various dermatoses [10,25]. Previous reports indicated that *CD32B* polymorphisms may be associated with systemic lupus erythematosus [26]. In mice [27], the inhibitory *CD32B* gene serves as an SLE suppressor and the inhibitory signaling cascade via this receptor is critical for the suppression of autoimmunity in the development of the disease. Furthermore, recent findings suggested that variations in FcγR expression and function could have profound effects on the modulation of B cell activity and immune phenotypes. Kasperkiewicz et al. [15] revealed that FcγRIV expression on neutrophils essentially contributed to autoantibody-induced tissue injury in a transfer model of a type of ABD, epidermolysis bullosa acquisita.

Interestingly, we failed to identify any statistically significant differences between specific genotypes of the aforementioned *CD16A* and *CD32A* polymorphisms and circulating autoantibodies levels in pemphigus and SABDs. However, we did reveal a relation between certain genotypes of *CD16A* and tissue-bound antibodies in pemphigus and SABDs, showing that the investigated SNP of *CD16A* probably induces direct reactions in the skin rather than a systemic response. Therefore, its pathogenic correlation has no direct connection with the level of anti-DSG1/DSG3 IgG nor anti-BP180/230 IgG in the serum of pemphigus and SABD patients, respectively.

Furthermore, the balance between SNPs involved in activating and inhibitory Fcγ receptor signaling may partially affect the development and progression of autoimmune diseases through the regulation of Th1 and Th2 cytokine levels. Our previous studies [5,28,29] demonstrated the role of IgG subclasses in a temporal analysis of humoral response in ABDs, indicating that *CD16A* polymorphisms (rs396991) are probably linked to the IgG1 subclass in pemphigus, and may therefore serve as a marker of chronic disease or remission.

According to our results, it may be postulated that *CD16A* polymorphisms are associated with an unspecific immune response in the skin at the molecular level in pemphigus and SABDs rather than directly with ABD phenotype expressions or manifestations. Therefore, FcR SNPs may be related to antibody class/subclass switching in ABDs, thereby determining the severity of the disease. However, obtained results suggested that this is a local process rather than a systemic reaction due to a lack of change in serum antibody levels. Our study potentially indicated that the SNP of *CD16A* is associated with autoantibody subclass changes, which may significantly affect the course of disease and perhaps help to diagnose respective ABDs. Defective signaling of *CD16* is a probable factor in the augmented reactivity of B cells in pemphigus and SABDs.

Scientific findings indicated a significant role of complement activation in ABD pathogenesis [30,31]. Kushner et al. [32] indicated complement inhibition as a therapeutic strategy in human BP. Our data, which revealed the genotype CA to be associated with C3 immunodeposition, suggested that rs396991 of *CD16A* may be a molecular element of local complement activation functioning to induce an inflammatory cascade and resulting in blister formation in BP. This probable mechanism of action was demonstrated previously in an animal model of BP [12], where mice deficient in CD16 were resistant to BP. CD16 deficiency is probably associated with a lack of neutrophil activation and subsequent secretion of proteolytic enzymes in damaged skin.

The role of antibodies and their isotypes in disease activity of pemphigus is well known [33,34]. Our results indicate that rs396991 of *CD16A* (AA genotype) is related to IgG1 immunodeposition, which could reveal a chronic stage of disease.

It should be noted that the identification of specific polymorphisms of *CD32A* may have therapeutic potential. Studies indicated that allelic polymorphisms in *CD32A* may correlate with the efficacy of immunoglobulin-based therapeutics according to their variable affinity for immunoglobulins. It was also suggested that the therapeutic response to rituximab (anti-CD20 monoclonal antibody) [35,36,37] is associated with the *CD32A* genotype, while FcγRs may have an effect on intravenous IgG-related immunomodulation [37].

Our findings suggest that *CD16* SNPs may contribute to pemphigus and SABD susceptibility in Caucasian populations and may thus serve as a pivotal candidate for a genetic risk marker in immunological profiling.

## 5. Conclusions

We conclude that *CD16A* and *CD32A* could predict clinical prognosis (Th2-mediated active stage or remission) in patients with pemphigus and SABD assessment of FcγR polymorphisms. Specific rs1801274 and rs396991 genotypes appear to not be directly related to circulating antibody production in the serum.

We postulate that rs396991 (*CD16A* polymorphism) may be of greater importance in ABD development, where (i) the genotype CA (rs396991) is probably associated with the complement activation pathway in SABDs (*p* = 0.0078; Chi-square test), and (ii) the genotype AA (rs396991) is probably associated with the IgG1-mediated response (chronic stage of disease) in pemphigus (*p* = 0.0121; Chi-square test).

FcR polymorphisms have a greater impact on tissue-bound antibodies detected with DIF than circulating antibodies in the serum of ABD patients.

## Figures and Tables

**Table 1:** Characteristics of the examined groups.

Parameter	SABDs Group	Pemphigus Group
**Numer of patients**	47	14
**Sex**	28 F; 19 M	9 F; 5M
**Mean age ± SD**	77.5 ± 12.4 (min.55; max. 95)	63.5 ± 16.8 (min. 35, max. 86)
**ELISA score (mean ± SD; RU/mL)**		
**Anti-DSG1 IgG**	NA	126.04 ± 93.48
**Anti-DSG3 IgG**	NA	54.79 ± 85.09
**Anti-BP180 IgG**	123.74 ± 82.53	NA
**Anti-BP230 IgG**	51.61 ± 73.81	NA

Abbreviations: SABDs—subepithelial autoimmune blistering disorders (including epidermolysis bullosa acquisita (EBA) and mucous membrane pemphigoid (MMP)); F—female; M—male; NA—not applicable; min.—minimum; max.—maximum.

**Table 2:** The distribution of various alleles and genotypes of rs396991 of *CD16A* and rs1801274 of *CD32A* in the examined population: SABDs and pemphigus.

**rs396991** **of *CD16A***	**SABDs (*n = 47*)** **n (*%*)**	**Pemphigus (*n = 14*) n (*%*)**	**OR (95% CI)**	***p*-Value**
*allele*				
*A*	64 (68.00)	18 (65.00)	1.18 (0.49–2.88)	0.8191
*C*	30 (32.00)	10 (35.00)	0.84 (0.34–2.05)	
*genotypes*				
*AA*	21 (44.68)	5 (36.00)	1.62 (0.48–5.46)	0.7593
*CA*	22 (46.81)	8 (57.00)	0.77 (0.24–2.47)	0.5541
*CC*	4 (8.51)	1 (7.00)	0.60 (0.10–3.63)	0.8699
*p*HW	0.5971	0.3604		
**rs1801274** **of *CD32A***	**SABDs (*n = 47*)** **n (*%*)**	**Pemphigus (*n = 14*) n (*%*)**	**OR (95% CI)**	***p-*Value**
*allele*				
*T*	43 (46.00)	16 (57.20)	0.63 (0.27–1.48)	0.3892
*C*	51 (54.00)	12 (42.80)	1.58 (0.67–3.71)	
*genotypes*				
*TT*	9 (19.15)	4 (28.60)	0.59 (0.15–2.32)	0.4716
*TC*	25 (53.19)	8 (57.10)	0.89 (0.37–2.99)	1.0000
*CC*	13 (27.66)	2 (14.30)	2.29 (0.45–11.68)	0.4832
*p*HW	0.6236	0.7821		

Abbreviations: SABDs—subepithelial autoimmune blistering disorders (including epidermolysis bullosa acquisita (EBA) and mucous membrane pemphigoid (MMP)); n—number of patients; OR—odds ratio; CI—confidence interval; *p*HW—Hardy–Weinberg *p*-value.

**Table 3:** Comparison of direct immunofluorescence results in relation to various genotypes of rs396991 of *CD16A* and rs1801274 of *CD32A* in SABD patients.

**rs396991**	**AA** **n (% of all)**	**CA** **n (% of all)**	**CC** **n (% of all)**	***p*-** **Value** **(Chi-Square Test)**
IgG −	21 (44.68%)	20 (42.55%)	4 (8.51%)	0.6674
IgG +	0 (0%)	1 (2.13%)	0 (0%)	
IgG ++	0 (0%)	1 (2.13%)	0 (0%)	
IgG1 −	19 (40.42%)	16 (34.04%)	4 (8.51%)	0.4001
IgG1 +	1 (2.13%)	5 (10.64%)	0 (0%)	
IgG1 ++	1 (2.13%)	0 (0%)	0 (0%)	
IgG1 +++	0 (0%)	1 (2.13%)	0 (0%)	
IgG4 −	3 (6.52%)	9 (19.56%)	0 (0%)	0.1077
IgG4 +	6 (13.04%)	2 (4.35%)	2 (4.35%)	
IgG4 ++	7 (15.22%)	4 (8.70%)	2 (4.35%)	
IgG4 +++	5 (10.87%)	6 (13.04%)	0 (0%)	
C3 −	6 (13.64%)	7 (15.91%)	0 (0%)	0.0078
C3 +	3 (6.82%)	2 (4.55%)	4 (9.09%)	
C3 ++	8 (18.18%)	9 (20.44%)	0 (0%)	
C3 +++	2 (4.55%)	3 (6.82%)	0 (0%)	
**rs1801274**	**TC** **n (% of all)**	**TT** **n (% of all)**	**CC** **n (% of all)**	***p*-** **Value**
IgG −	25 (53.19%)	8 (17.02%)	12 (25.53%)	0.1388
IgG +	0 (0%)	1 (2.13%)	0 (0%)	
IgG++	0 (0%)	0 (0%)	1 (2.13%)	
IgG1 −	21 (44.68%)	7 (14.89%)	11 (23.40%)	0.6179
IgG1 +	3 (6.38%)	2 (4.26%)	1 (2.13%)	
IgG1 ++	1 (2.13%)	0 (0%)	0 (0%)	
IgG1 +++	0 (0%)	0 (0%)	1 (2.13%)	
IgG4 −	8 (17.38%)	2 (4.35%)	2 (4.35%)	0.9140
IgG4 +	4 (8.70%)	2 (4.35%)	4 (8.70%)	
IgG4 ++	7 (15.22%)	3 (6.52%)	3 (6.52%)	
IgG4 +++	6 (13.04%)	2 (4.35%)	3 (6.52%)	
C3 −	7 (15.91%)	4 (9.08%)	2 (4.55%)	0.4938
C3 +	4 (9.08%)	2 (4.55%)	3 (6.82%)	
C3 ++	10 (22.73%)	2 (4.55%)	5 (11.36%)	
C3 +++	2 (4.55%)	0 (0%)	3 (6.82%)	

Abbreviations: SABDs—subepithelial autoimmune blistering disorders (including epidermolysis bullosa acquisita (EBA) and mucous membrane pemphigoid (MMP)); n—number of patients; Ig—immunoglobulin; C3—complement component 3; “−” negative reaction; “+” positive reaction; “++” strong positive reaction; “+++” very strong positive reaction.

**Table 4:** Comparison of direct immunofluorescence results in relation to various genotypes of rs396991 of *CD16A* and rs1801274 of *CD32A* in pemphigus patients.

**rs396991**	**AA** **n (% of all)**	**CA** **n (% of all)**	**CC** **n (% of all)**	***p*-Value** **(Chi-Square Test)**
IgG −	1 (8.33%)	5 (41.68%)	1 (8.33%)	0.2397
IgG +	3 (25.00%)	1 (8.33%)	0 (0%)	
IgG ++	1 (8.33%)	0 (0%)	0 (0%)	
IgG1 −	1 (9.09%)	4 (36.37%)	0 (0%)	0.0121
IgG1 +	3 (27.27%)	2 (18.18%)	0 (0%)	
IgG1 ++	0 (0%)	0 (0%)	1 (9.09%)	
IgG1 +++	0 (0%)	0 (0%)	0 (0%)	
IgG4 −	0 (0%)	0 (0%)	0 (0%)	0.2106
IgG4 +	0 (0%)	3 (21.43%)	0 (0%)	
IgG4 ++	4 (28.57%)	3 (21.43%)	0 (0%)	
IgG4 +++	1 (7.14%)	2 (14.29%)	1 (7.14%)	
C3 −	0 (0%)	2 (16.67%)	0 (0%)	0.2263
C3 +	3 (25.00%)	1 (8.33%)	1 (8.33%)	
C3 ++	1 (8.33%)	4 (33.34%)	0 (0%)	
C3 +++	0 (0%)	0 (0%)	0 (0%)	
**rs1801274**	**TC** **n (%)**	**TT** **n (%)**	**CC** **n (%)**	***p*-Value**
IgG −	5 (41.66%)	2 (16.67%)	0 (0%)	0.0969
IgG +	0 (0%)	2 (16.67%)	2 (16.67%)	
IgG++	1 (8.33%)	0 (0%)	0 (0%)	
IgG1 −	3 (27.28%)	2 (18.18%)	0 (0%)	0.4529
IgG1 +	2 (18.18%)	1 (9.09%)	2 (18.18%)	
IgG1 ++	1 (9.09%)	0 (0%)	0 (0%)	
IgG1 +++	0 (0%)	0 (0%)	0 (0%)	
IgG4 −	0 (0%)	0 (0%)	0 (0%)	0.1497
IgG4 +	1 (7.14%)	2 (14.29%)	0 (0%)	
IgG4 ++	5 (35.70%)	0 (0%)	2 (14.29%)	
IgG4 +++	2 (14.29%)	2 (14.29%)	0 (0%)	
C3 −	1 (8.33%)	1 (8.33%)	0 (0%)	0.4779
C3 +	2 (16.67%)	1 (8.33%)	2 (16.67%)	
C3 ++	3 (25.00%)	2 (16.67%)	0 (0%)	
C3 +++	0 (0%)	0 (0%)	0 (0%)	

Abbreviations: n—number of patients; Ig—immunoglobulin; C3—complement component 3; “−” negative reaction; “+” positive reaction; “++” strong positive reaction; “+++” very strong positive reaction.

**Table 5:** The comparison of anti-BP180 IgG and anti-BP230 IgG levels in relation to various alleles of rs1801274 and rs396991 in SABD patients.

**SABDs**	**Group/** **Allele**	**n**	**Mean ± SD**	**Median**	**Min.**	**Max.**	***p-*Value**
			(RU/mL)	(RU/mL)	(RU/mL)	(RU/mL)	
**rs1801274 of *CD32A***							
Anti-BP180 IgG	T	41	118.50 ± 79.85	84.63	5.48	200	0.7716
	C	49	128.11 ± 84.43	172.67	5.48	200	
Anti-BP230 IgG	T	39	56.91 ± 76.39	11.05	0.49	200	0.7824
	C	47	47.20 ± 71.30	13.37	0.49	200	
**rs396991 of *CD16A***							
Anti-BP180 IgG	A	61	125.13 ± 84.70	117.22	5.48	200	0.9863
	C	29	120.79 ± 77.58	92.98	16.27	200	
Anti-BP230 IgG	A	57	54.57 ± 75.21	13.37	0.48	200	0.8845
	C	29	45.78 ± 70.55	13.37	1.56	200	

Abbreviations: SABDs—subepithelial autoimmune blistering disorders (including epidermolysis bullosa acquisita (EBA) and mucous membrane pemphigoid (MMP)); n—number of patients; SD—standard deviation; min.—minimum; max.—maximum.

**Table 6:** The comparison of anti-DSG1 IgG and anti-DSG3 IgG levels in relation to various alleles of rs1801274 and rs396991in pemphigus patients.

**Pemphigus**	**Group/** **Allele**	**n**	**Mean ± SD**	**Median**	**Min.**	**Max.**	***p-*Value**
			(RU/mL)	(RU/mL)	(RU/mL)	(RU/mL)	
**rs1801274 of *CD32A***							
Anti-DSG1 IgG	T	16	122.42 ± 94.53	175.03	0.86	200	0.8017
	C	12	130.85 ± 91.77	183.34	2.68	200	
Anti-DSG3 IgG	T	16	50.57 ± 80.64	7.45	0.76	200	0.3713
	C	12	60.42 ± 90.47	10.81	2.14	200	
**rs396991 of *CD16A***							
Anti-DSG1 IgG	A	18	127.46 ± 90.99	175.03	0.86	200	0.9437
	C	10	123.48 ± 97.94	175.03	0.86	200	
Anti-DSG3 IgG	A	18	55.38 ± 85.82	8.29	0.86	200	0.8691
	C	10	53.73 ± 83.67	8.85	0.76	200	

Abbreviations: n—number of patients; SD—standard deviation; min.—minimum; max.—maximum.
